# Effect of Different Mouthwashes on the Surface Microhardness and Color Stability of Dental Nanohybrid Resin Composite

**DOI:** 10.3390/polym15040815

**Published:** 2023-02-06

**Authors:** Tamer M. Hamdy, Ali Abdelnabi, Maha S. Othman, Rania E. Bayoumi, Rasha M. Abdelraouf

**Affiliations:** 1 Restorative and Dental Materials Department, Oral and Dental Research Institute, National Research Centre (NRC), Giza 12622, Egypt; 2 Operative Dentistry Department, Faculty of Dentistry, Cairo University, Cairo 11553, Egypt; 3 Biomaterials Department, Faculty of Dentistry (Girls), Azhar University, Cairo 11754, Egypt; 4 Biomaterials Department, Faculty of Dentistry, Cairo University, Cairo 11553, Egypt

**Keywords:** polymer composites, mouthwashes, microhardness, color stability, resin composite, bleaching, green tea, chlorohexidine

## Abstract

Background: Surface microhardness and color stability of dental restorative material should be sustained throughout its functional lifetime to maintain the esthetic quality of the restoration. However, the frequent application of mouthwash may affect their surface microhardness and color stability. The aim of this study was to evaluate the effects of different types of mouthwashes with different contents on surface microhardness and color stability of dental nanohybrid resin-based composite. Methods: Disc specimens of nanohybrid resin composite (Luna Nano-Hybrid Composite) were prepared according to manufacturing instructions; specimens were incubated for 24 h in three types of mouthwash (Chlorohexidine, Listerine Green Tea, and Colgate Optic White Whitening Mouthwash). Artificial saliva was used as a control group. Surface microhardness was evaluated using Vickers microhardness device. Color stability after and before immersion in the different mouthwashes was evaluated using extra-oral spectrophotometer; the values of color change (ΔE_00_) were subsequently calculated. Data were analyzed using one-way ANOVA and post hoc test (*p* ≤ 0.05). Results: There was no significant difference between microhardness of resin composite immersed in artificial saliva, CHX, and Green Tea mouthwashes (78.5, 78.4, and 73.5, respectively) (*p* ≥ 0.1), while the bleaching mouthwash led to the lowest microhardness of resin composite, with significant difference compared to the three previous immersion media (*p* = 0.002). Moreover, there were significant differences in the color changes (ΔE_00_) of resin composite exposed to the various immersion media (*p* = 0.0001). Conclusions: The bleaching mouthwash led to a significant reduction in nanohybrid resin composite’s microhardness compared to the chlorohexidine and Green Tea containing mouthwashes. The resin composite’s color change was accepted in bleaching mouthwash but unaccepted in chlorohexidine and Green Tea containing mouthwashes.

## 1. Introduction

Resin-based dental composites are currently the preferred restorative materials for direct anterior as well as posterior dental esthetic restorations [1]. The continuous development of nanotechnology in the field of restorative dentistry provides a challenging achievement in the synthesis of the resin composite, including nano-filled and nanohybrid resin composites [2]. Nanohybrid resin composites are the most propitious due to their improvement in the distribution of fillers into matrix by incorporation of both traditional submicron particles, together with emerging nanoparticles, which, in turn, provides enhanced mechanical and optical properties [3,4,5,6,7]. Currently, nanohybrid resin composite is employed as a suitable direct posterior dental restorative material that has expressed adequate durability in clinical investigations [8,9]. Microhardness is influenced not only by the composition of the resin composite, but also by the type of polymerization employed during clinical work [10]. Low surface microhardness permits plaque accumulation on the surface of composite restorations, which may be responsible for gingivitis, especially in proximal resonation, and, in addition, staining and recurrent caries. Therefore, sufficient surface microhardness is a significant feature of the dental restorative materials [11,12]. Moreover, nanohybrid resin composite provides a substantial esthetic potential for direct esthetic restoration [13]. Enhanced optical properties, together with color stability, are important properties to provide esthetic dental restorations [6,14].

Resin composite restorations are subjected to different types of stains in the oral cavity [15,16]. Discoloration is generally divided into internal and external discoloration; internal discoloration of resin composites is produced by physical and chemical reactions in the innermost layers of the resin composite, while the external discoloration mainly arises from consumption of coloring agents from different sources, such as food, beverages, smoking, and frequent application of mouthwashes [17,18].

Oral mouthwash treatment is recommended in specific conditions, such as in treatment of periodontal diseases, halitosis, and as a preventive measure during orthodontic treatments and whitening mouth rinses [19]. The frequent use of mouthwash has a potential to adversely affect the surface properties of the resin composites and cause a discoloration for both teeth and restorative materials [20,21,22].

A varied diversity of commercial synthetic mouthwashes is available; Chlorhexidine (CHX) is the most used mouthwash in competing dental plaque and is described as the gold standard antiseptic mouthwash. Even though CHX has been reported to be very effective in reducing bacterial dental plaque, it yields teeth staining and causes discoloration in both teeth and restorations [23,24]. Using Green Tea containing mouthwash has a comparable bactericidal effect to that of CHX, but its effect on resin composite surface properties and color stability is still unknown [25,26]. Recently, home bleaching procedure has become a popular process in removing stains to obtain an esthetic appearance of natural teeth. Home bleaching mouthwashes involve the application of hydrogen peroxide agents as active ingredients [27].

The existing restorations and natural teeth during home bleaching process are subjected to bleaching agents for a long duration, which may cause a serious effect on the existing resin-based restorative materials [28]. There are limited data regarding the effect of application of home bleaching using whitening mouthwash on the surface microhardness and coloring stability of resin-based dental composite. Consequently, the present study investigated the effect of three different types of mouthwashes on the surface microhardness and color stability of nanohybrid resin composite. Color changes of resin composite after immersion in artificial saliva and various mouthwashes (MW) were analyzed across red–green axis (Δa), yellow–blue axis (Δb), and lightness (ΔL).

The null hypotheses tested were as follows: (1) no significant differences exist between the tested mouthwashes with respect to surface microhardness and (2) no significant differences exist between the tested mouthwashes in terms of color stability of the resin composite.

## 2. Materials and Methods

**Figure 1:**
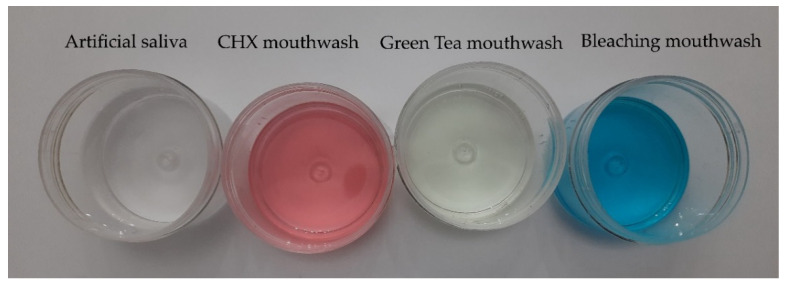
Artificial saliva and mouthwashes used in the study.

Nanohybrid resin composite (Luna nanohybrid composite for anterior/posterior restorations, Lot. Number: 2110140, SDI limited, Bayswater, VIC, Australia) was treated with three different types of mouthwashes (Chlorohexidine-based mouthwash, Listerine Green Tea mouthwash, and Colgate Optic White Whitening Mouthwash) and artificial saliva as the control group, Figure 1. Shade A2 was used as the standard. Ingredients regarding these materials are expressed in Table 1.

### 2.1. Specimen Preparation

A total of 80 specimens were prepared using Teflon mold with 8 mm diameter and 1 mm thickness for production of disc-shaped specimens [29,30]. After resin composite was filled into the Teflon mold, a transparent celluloid strip (Mylar strip; SS White Co. Philadelphia, PA, USA) and glass plate covered it and light pressure was applied after that to remove any excess material and obtain a highly smoothed surface. The specimens were cured according to the manufacturer’s instructions for 40 s using a light emitting diode (LED) curing unit (LED device Mini LED, Satelec, Acteon, France), with wavelength 400–500 nm and the light intensity was 1000 mW/cm^2^. The distance between the specimens and light source and the sample was standardized by using a glass slide (1 mm thickness). After curing procedure, polishing of the specimens on both sides was performed using composite polishing kit (Shofu Composite Polishing Kit, Shofu Dental GmbH, Ratingen, Germany). Specimens were then stored in distilled water at 37 °C for 24 h to ensure post-polymerization.

### 2.2. Specimen Grouping

The specimens were randomly distributed into 8 groups according to each tested mouthwash. In each group, 10 specimens (n = 10) were randomly selected for surface microhardness analysis and the remaining 10 (n = 10) for color stability investigation. The specimens were immersed in 5 mL of the tested mouthwash at 37 °C for 24 h and they were subjected to the final testing.

- Group 1: specimens immersed in artificial saliva (control).
- Group 2: specimens immersed in CHX mouthwash.
- Group 3: specimens immersed in Green Tea mouthwash.
- Group 4: specimens immersed in Colgate Optic White mouthwash.

The pH value of each mouthwash was measured by pH meter (Jenway 3510 bench pH meter, Fisher Scientific, Loughborough, UK). The parameters determined in the current study were surface microhardness and color stability.

### 2.3. Microhardness Test

Surface microhardness for each specimen was determined using Digital Vickers hardness tester (NEXUS 400TM, INNOVATEST, model no. 4503, Maastricht, The Netherlands). Five indentations were made within 15 s dwell time at load 100 g at 20× magnification [31]. The mean surface microhardness value for each specimen was calculated.

### 2.4. Color Stability

For each specimen, color parameter was measured before immersion in the storage mouthwash as baseline color (T_0_). After the immersion period, the final (T_f_) color measurements were performed for each specimen, using extra-oral spectrophotometer (Cary 5000, Agilent Technologies, Santa Clara, CA, USA). The color difference (ΔE_00_) was determined using the CIEDE2000 following the Commission International de l’Eclariage (CIE) L*a*b* system compared to CIE standard illuminant D65 against a black background [32]. The calculation is based on the equation:

(1) $$\Delta E_{00}=\sqrt{{\left(\frac{\Delta L\prime }{K_{L}\cdot S_{L}}\right)}^{2}+{\left(\frac{\Delta C\prime }{K_{C}\cdot S_{C}}\right)}^{2}+{\left(\frac{\Delta H\prime }{K_{H}\cdot S_{H}}\right)}^{2}+(R_{T}(\frac{\Delta C\prime }{K_{C}\cdot S_{C}})(\frac{\Delta H\prime }{K_{H}\cdot S_{H}}))}$$

where ∆L: lightness difference, ∆C: chroma difference, ∆H: hue difference, and S_L_, S_C_, S_H_, K_L_, K_C_, and K_H_: constant coefficients.

Measurements were performed at wavelengths ranging from 380 to 780 nm at 1 nm intervals. The calculation of ΔE_00_ was achieved using the Excel spreadsheet enactment of the CIEDE2000 color difference formula [32]. The resultant values were then correlated to perceptibility and acceptability thresholds, which are 0.8 and 1.8, respectively [33].

### 2.5. Statistical Analysis

Statistical analyses of the data were performed using the Statistical Package for the Social Sciences, version 26 for windows (SPSS, IBM, New York, NY, USA). The comparison of the mean values between groups for the pH changes, microhardness, and color change (ΔE_00_) was performed using one-way ANOVA and post hoc test. The significance level was set at *p* ≤ 0.05.

## 3. Results

The pH of the artificial saliva and the bleaching mouthwash was neutral (7 and 7.1, respectively), with no significant difference between them (*p* = 0.24), while the pH of CHX and Green Tea mouthwashes was acidic (3.9 and 4.2, respectively), Table 2.

There was no significant difference between microhardness of resin composite immersed in artificial saliva, CHX, and Green Tea mouthwashes (78.5, 78.4, and 73.5, respectively), (*p* ≥ 0.1), while the bleaching mouthwash led to the lowest microhardness of resin composite, with significant difference compared to the three previous immersion media (*p* = 0.002), Table 3.

There were significant differences in the color changes (ΔE_00_) of resin composite exposed to the various immersion media (*p* = 0.0001), Table 3. The lowest color change was for resin composite immersed in artificial saliva (ΔE_00_ = 0.9), followed by bleaching mouthwash (ΔE_00_ = 1.6), then CHX mouthwash (ΔE_00_ = 2.6). The highest color change was after immersion in Green Tea mouthwash (ΔE_00_ = 3.2).

The color changes in resin composite immersed in artificial saliva (ΔE_00_ = 0.9) were just above the perceptibility threshold (ΔE_00_ = 0.8), while the color changes of resin composite after bleaching mouthwash (ΔE_00_ = 1.6) were above the perceptibility threshold but within accepted range (acceptability threshold ΔE00 = 1.8). On the other hand, the color change of CHX mouthwash was unaccepted (ΔE_00_ = 2.6), exceeding the acceptability threshold. The highest unacceptable color change was reported for resin composite exposed to Green Tea mouthwash (ΔE_00_ = 3.2).

**Figure 2:**
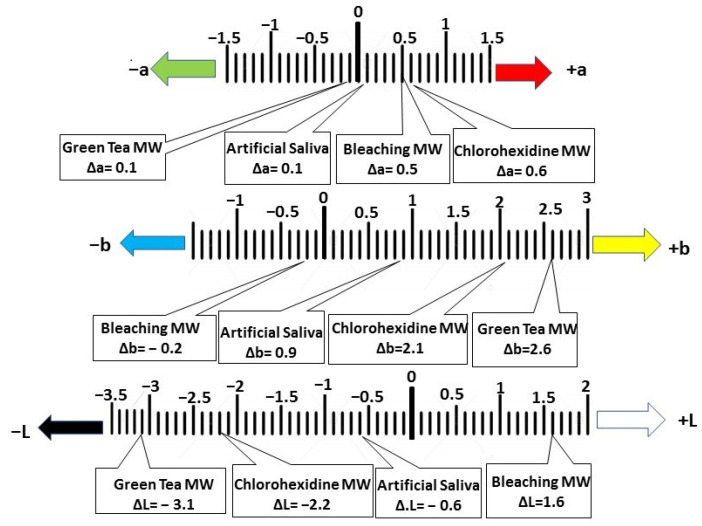
Color changes in resin composite after immersion in artificial saliva and various mouthwashes across red–green axis (Δa), yellow–blue axis (Δb), and lightness (ΔL).

Color changes in resin composite after immersion in artificial saliva and various mouthwashes (MW) were analyzed across red–green axis (Δa), yellow–blue axis (Δb), and lightness (ΔL), Figure 2.

Hue change across red–green axis (Δa) showed that the immersion in artificial saliva, bleaching, and CHX mouthwashes led to a reddish discoloration of resin composite (Δa = 0.1, 0.5, and 0.6, respectively). On the other hand, Green Tea mouthwash led to a slight green discoloration (Δa = −0.1).

Hue change across yellow–blue axis (Δb) showed that the artificial saliva, CHX, and Green Tea mouthwashes increased the yellowish discoloration of resin composite (Δb = 0.9, 2.1, and 2.6, respectively). Meanwhile, the bleaching mouthwash led to a reduction in the yellow content and a slight shifting towards the blue (Δb = −0.2).

Changes in the lightness or darkness of resin composite (ΔL) revealed reduction in the value (represented by the negative ΔL values) after immersion in the artificial saliva, CHX, and Green Tea mouthwashes (ΔL = −0.6, −2.2, and −3.1, respectively). This means that the resin composite became darker. In contrast, immersion in bleaching mouthwash led to a positive ΔL value (ΔL = 1.6), indicating that the resin composite became lighter.

## 4. Discussion

Polymers are commonly used in the dental field [34,35,36,37,38,39]. Dental resin composite is a common polymer-based esthetic restorative material. Yet, its polymeric organic matrix may lead to some challenges, which may affect its durability [40,41,42]. Among these drawbacks is the color stability. Discoloration of resin composite may occur by time due to internal or external sources. Internal discoloration may occur due to aging of resin composite components themselves, such as residual monomer and activator-initiator system [30]. External discoloration may occur due to food intake, beverages, and mouthwashes. The frequent use of mouthwashes may be considered a double-edged weapon. Their anti-inflammatory, antimicrobial, and analgesic properties enhance and regulate periodontal health. On the other hand, some ingredients in mouthwash may soften the organic resinous matrix and discolor it [19,43]. Spectrophotometer instruments are used to measure the color to attain precise, reliable, and repeatable outcomes. It was employed for color measurements throughout the reflection or transmission of an observed entity and is commonly used to investigate the color changes in restorative materials [44].

In this study, several mouthwashes were used. The CHX-containing mouthwash was used as it is considered the gold standard due to its proven potent antimicrobial activity. Yet, its discoloration and softening effect to resin composite was reported in the dental literature. Green Tea containing mouthwash represents one of the products containing natural ingredients, which could be a competitor for CHX [45].Yet, its effect on microhardness and color was not assessed. Another category of mouthwashes which have been launched in the dental market are the bleaching group. Yet, the effect of their peroxides on the resinous matrix is questionable. In this research, resin composite stored in artificial saliva used as control and the effect of these different mouthwashes was investigated on the resin composite’s color and microhardness. The storage time in this study was one day, which simulates two years clinically (2 min/day) [46].

The pH is considered one of the factors which may affect the organic resin matrix. Thus, it was measured in this study. The pH of the artificial saliva was adjusted to simulate the pH of the average natural neutral saliva [47]. The pH of the bleaching mouthwash was also neutral; in contrast, the pH of CHX and Green Tea mouthwashes was acidic. This agreed with previous studies, which reported the great variation in pH of the various mouthwashes from being acidic to alkalinity [48]. This large variation in pH values may be attributed to their compositional difference. It should be noted that some ingredients of the mouthwashes are unstable and may need certain pH values to prolong their shelf life without decomposition. For example, the Green Tea contains catechins, which have multiple positive biological activities that decompose within a few minutes in alkaline medium (pH 8). On the other hand, it shows high stability in acidic solution (pH 4). This might explain the pH 4 of the Green Tea containing mouthwash [49]. Similarly, CHX gluconate requires acidic pH solution to prevent its degradation during storage, increasing its life span [50,51]. This might explain the deviation from neutrality, which simulates the normal saliva with pH range of 6.2–7.6 [47]. It was reported that acidity, especially the critical pH = 5.5, may lead to demineralization of tooth structure [52,53]. Yet, most mouthwashes are applied only in an average time of 30 s [54]. However, care should be taken to avoid the possibility of occurrence of enamel erosion by low pH mouthwashes, which was reported previously [55]. In vivo studies proposed that 30 s could be enough time for an interaction between the acid and tooth structure, while in vitro studies reported several minutes [56]. Thus, it was recommended that mouthwashes with low pH should not be prescribed in the long term and never be used before brushing. Generally, patients are advised not to brush their teeth immediately after any acidic beverages and, within 10 min, in addition, a fluoride-containing mouthwash was suggested to be used. Thus, the fluoride content in the mouthwash could be considered as repairable source to any demineralized effect of mouthwash itself [57]. However, immediate brushing was not recommended after using acidic mouthwash for safety [55]. The effect of these mouthwashes on resin composite may be different from that of tooth structure due to the great variation in the chemical composition. The tooth structure is composed mainly of hydroxyapatite crystals, with its minerals being very sensitive to acidic attacks and resultant demineralization. On the other hand, the resin composite is composed of mainly inorganic fillers and organic matrix, which is considered the weaker phase which could be affected by chemicals, leading to degradation and decrease in the mechanical properties. In this study, although the pH of the bleaching mouthwash was neutral, it led to the significant reduction in resin composite hardness. This may be attributed to its hydrogen peroxide content, which belongs to the peroxide family containing ʺoxygen-oxygenʺ single bond. The hydrogen peroxide is a strong oxidizing agent due to its chemical structure and unpaired electrons. The hydrogen peroxide is an unstable molecule and easily decomposes due to its unstable peroxide bond. This induces the separation of the polymeric chain of the composite resin, weakening its double bonds, resulting in a softer surface affecting its hardness [58].

Although CHX and Green Tea mouthwashes were acidic, there was no significant difference between their microhardness of resin composite and that immersed in artificial saliva. This acidic pH was not considered a significant factor affecting the surface degradation of the resin composite [59]. Therefore, the chemical composition of the mouthwash could be considered a more effective factor affecting the resin composite microhardness more than its pH. The chemical composition of the mouthwash also affects the color change in resin composite. In addition, pigments play a major role in resin composite discoloration. Szalewski et al. observed that low pH diet may also increase the erosive effect on dental tissues as well resin composites [60].

The highest color change was after immersion in Green Tea mouthwash (ΔE_00_ = 3.2). This may be attributed to the tannin content of Green Tea, which is a polyphenol compound that is yellow to brown in color, which may cause the resin composite to discolor and become darker [61]. Green Tea also contains chlorophylls, which are pigments present in fresh leaves but that degrade easily. In the presence of acids, a reaction occurs between chlorophylls and acids producing pheophytin, an olive-brown solid [62]. This might explain why the resin composite became slightly greenish, yellowish, and darker after immersion in Green Tea.

The color change in resin composite exposed to CHX mouthwash was below that immersed in Green Tea but still unaccepted (ΔE_00_ = 2.6). This agreed with previous study, which observed the discoloring effect of CHX-containing mouthwashes [63,64]. It was reported that CHX gluconate led to yellow brown stains on the surface of restorative materials, as the CHX gluconate molecule could release parachloranilin with metal sulfide formation [64]. This may clarify why there was a shift in resin composite hue toward yellow along yellow–blue axis (Δb) and in value towards black along white–black axis (ΔL).

It was observed that the hue of the resin composite had the tendency to shift towards the hue of the mouthwash itself along red (+a)–Green (−a) axis and yellow (+b)–blue (−b) axis. For example, the CHX mouthwash was red in color and the resin composite showed deviation towards red (Δa = 0.6). The Green Tea mouthwash was green and the resin composite showed deviation towards green (Δa = −0.1). The bleaching mouthwash was blue and the resin composite showed deviation towards blue (Δb = −0.2). This may be attributed to the effect of coloring agents, which are added to enhance the mouthwashesʼ appearance [65]. Thus, excessive addition of such dyes could be minimized by the manufacturers.

The bleaching mouthwash was the only immersion media that led to a whitening effect, with a positive ΔL value (ΔL = 1.6). This may be attributed to the effect of hydrogen peroxide molecules, which, as mentioned previously, decompose easily, especially in the presence of metal ions, enzymes, or increased temperature, producing free radicals, which are unstable oxidants. Having unpaired electrons, they have a tendency to lower their energy and become stable. Therefore, these free radicals react with pigment molecules, resulting in degradation into simpler products [66]. In this study, the color change in resin composite after immersion in bleaching mouthwash (ΔE_00_ = 1.6) and artificial saliva (ΔE_00_ = 0.9) was perceptible but within the accepted range. This agreed with previous studies which reported perceptible color change in resin composite stored in artificial saliva [30,67]. This may be due to sorption of salivary components, as reported in a previous study [67]. The present in vitro study has some limitations, such as the usual brushing mechanism, in addition to the continuous normal washing effect of the saliva, which may reduce the staining effect. Therefore, further studies are recommended putting into consideration the capability of repolishing procedure to simulate the normal brushing. Moreover, in vivo studies are required to evaluate the washing effect of saliva and the effect of their enzymatic action on the staining deposition.

## 5. Conclusions

Within the limitations of this study, it can be concluded that the surface microhardness of the nanohybrid resin composite was affected by mouthwashes’ chemical compositions more than their pH. Although being neutral, the bleaching mouthwash led to significant reduction in resin composite’s microhardness compared to the acidic CHX and Green Tea containing mouthwashes. There were perceptible color changes after the frequent of mouthwashes simulating two years of clinical service; yet, these were accepted in bleaching mouthwash but unaccepted in CHX and Green Tea containing mouthwashes.

## Figures and Tables

**Table 1:** Ingredients of different studied materials.

Mouthwashes	Manufacturer	Composition
Artificial Saliva	Sigma, Sigma-Aldrich CO., Missouri City, TX, USA	Potassium chloride, sodium bicarbonate, sodium phosphate, potassium thiocyanate, and lactic acid
Oradex antibacterial mouthwash	Cavico Sdn Bhd., Selangor, Malaysia	Chlorhexidine gluconate (0.12%)
LISTERINE^®^ Green Tea	Johnson and Johnson S.p.A, Rome, Italy	Aqua, Propylene Glycol, Sorbitol, poloxamer 407, Sodium Lauryl Sulfate, Sodium Saccharin, Aroma, Eucalyptol, Benzoid Acid, Sodium Benzoate, Methyl Salicylate, thymol, Sodium Fluoride, Menthol, Sucralose, Camellia Sinensis Leaf Extracts, Caffeine, CI 47005, CI 42053, contains sodium fluoride (220 ppm F)
Colgate Optic White, Whitening Mouthwash	Colgate-Palmolive Co., New York, NY, USA	Water, Glycerin, Propylene Glycol, Sorbitol, Hydrogen Peroxide, Polysorbate 20, Sodium Acrylates/Methacryloylethyl Phosphate Copolymer, Phosphoric Acid, Citric Acid, Flavor, PVM/MA Copolymer, Sodium Saccharin

**Table 2:** The pH of artificial saliva and different mouthwashes.

	Artificial Saliva	Chlorhexidine Mouthwash	Green Tea Mouthwash	Bleaching Mouthwash	*p* Value
pH	7 ^a^ ± 0.04	3.9 ^c^ ± 0.1	4.2 ^b^ ± 0.07	7.1 ^a^ ± 0.1	*p* ≤ 0.006 *

Mean with different letters indicate statistically significant difference, *: significant (*p* < 0.05).

**Table 3:** The pH of artificial saliva and different mouthwashes.

	Artificial Saliva	Chlorhexidine Mouthwash	Green Tea Mouthwash	Bleaching Mouthwash	*p* Value
Microhardness of Resin Composite	78.5 ^a^ ± 1.4	78.4 ^a^ ± 0.7	73.5 ^a^ ± 2.1	67.4 ^b^ ± 2.8	*p* = 0.002
Color Change (ΔE_00_) of Resin Composite	0.9 ^a^ ± 0.04	2.6 ^c^ ± 0.08	3.2 ^d^ ± 0.1	1.6 ^b^ ± 0.07	*p* = 0.0001 *

Mean with different letters in the same row indicate statistically significant difference, *: significant (*p* < 0.05).

## Data Availability

The data presented in this study are available on request from the corresponding author.
